# NT-proBNP: Is It a More Significant Risk Factor for Mortality Than Troponin T in Incident Hemodialysis Patients?

**DOI:** 10.1097/MD.0000000000000241

**Published:** 2014-12-12

**Authors:** Hyung Jung Oh, Mi Jung Lee, Hye Sun Lee, Jung Tak Park, Seung Hyeok Han, Tae-Hyun Yoo, Yong-Lim Kim, Yon Su Kim, Chul Woo Yang, Nam-Ho Kim, Shin-Wook Kang

**Affiliations:** From the Department of Internal Medicine, College of Medicine, Brain Korea 21 for Medical Science, Severance Biomedical Science Institute, Yonsei University, Seoul, Korea (HJO, MJL, JTP, SHH, T-HY, S-WK); Department of Internal Medicine, Kyungpook National University School of Medicine, Daegu, Korea (Y-LK); Department of Internal Medicine, Seoul National University of Medicine, Seoul, Korea (YSK); Department of Internal Medicine, Seoul St. Mary's Hospital, College of Medicine, Seoul, Korea (CWY); Department of Medicine, Chonnam National University Medical School, Gwangju, Korea (N-HK); and Department of Biostatistics, Yonsei University College of Medicine, Seoul, Korea (HSL).

## Abstract

Numerous studies have demonstrated that cardiac biomarkers are significant predictors of cardiovascular (CV) and all-cause mortality in ESRD patients, but most of the studies were retrospective or included small numbers of patients, only prevalent dialysis patients, or measured 1 or 2 biomarkers. This study was to analyze the association between 3 cardiac biomarkers and mortality in incident HD patients. A prospective cohort of 864 incident HD patients was followed for 30 months. Based on the median values of baseline NT-proBNP, cTnT, and hsCRP, the patients were divided into “high” and “low” groups, and CV and all-cause mortality were compared between each group. Additionally, time-dependent ROC curves were constructed, and the NRI and IDI of the models with various biomarkers were calculated. The CV survival rates were significantly lower in the “high” NT-proBNP and cTnT groups compared to the corresponding “low” groups, while there was no significant difference in CV survival rate between the 2 hsCRP groups. However, all-cause mortality rates were significantly higher in all 3 “high” groups compared to each lower group. In multivariate analyses, only Ln NT-proBNP was found to be an independent predictor of mortality. Moreover, NT-proBNP was a more prognostic marker for mortality compared to cTnT. In conclusion, NT-proBNP is the biomarker that results in the most added prognostic value on top of traditional risk factors for CV and all-cause mortality in incident HD patients.

## Abstract 2

Supplemental Digital Content is available in the text

## INTRODUCTION

Cardiovascular disease (CVD) is prevalent, and is the leading cause of morbidity and mortality in patients with end-stage renal disease (ESRD).^1^ Among various CVDs, left ventricular hypertrophy (LVH) is the most frequent CV manifestation,^2,3^ present in more than 70% of incident ESRD patients, and it has been shown to increase the risk for cardiac ischemia, LV dysfunction, and sudden cardiac death.^4,5^ However, many dialysis patients are asymptomatic.^6^ Therefore, the identification of ESRD patients at high risk of CVD is important in order to expedite aggressive treatment and to improve patient outcomes.

Traditional risk factors for CVD, such as advanced age, hypertension, diabetes, and dyslipidemia, frequently coexist in ESRD patients,^7^ but they cannot fully account for the high prevalence of CVD in these patients; therefore, research must be performed to create better and easier tools for CVD risk stratification in this population.^8^ Recently, several biochemical markers, such as B-type natriuretic peptide (BNP), N-terminal proBNP (NT-proBNP), cardiac troponin T (cTnT), and I (cTnI), and high-sensitivity C-reactive protein (hsCRP), have received attention from researchers as potential candidates to assist with risk stratification.^8–14^

BNP belongs to a family of vasopeptide hormones and is secreted in prohormone form (proBNP) from the LV in response to wall stretch of the ventricles.^15–17^ In the circulation, proBNP is cleaved into the active C-terminal fragment and the biologically inactive NT-proBNP.^17^ The increase in BNP and NT-proBNP concentrations is associated with abnormal LV structure and function.^18,19^ Meanwhile, cTnT and cTnI are components of the contractile apparatus of the heart muscle and are released into the circulation after myocardial necrosis.^8,20,21^ In addition, accumulating evidence has shown that myocardial ischemia is closely linked with elevated levels of cTnT and cTnI.^20,21^ These 2 sorts of cardiac biomarkers have significant prognostic value for CV and all-cause mortality not only in the general population but also in patients with specific diseases, including ESRD.^8,10,11,22,23^

Uremia-related nontraditional risk factors, including inflammation and oxidative stress, have been implicated in the pathogenesis of CVD in dialysis patients.^24–27^ Accordingly, a number of previous studies investigated the association of hsCRP, which is thought to be a biomarker for inflammation, with the clinical outcomes in patients with ESRD and found that there was a correlation between hsCRP levels and mortality in these patients.^11,14,27,28^

Even though numerous previous studies have revealed that cardiac and inflammatory biomarkers are significant predictors of CV and all-cause mortality in ESRD patients, the majority were retrospective or included small numbers of patients, only examined prevalent dialysis patients, studied ESRD patients with different ethnicities or dialysis modalities, or only measured 1 or 2 biomarkers.^9–11,23^ In the present study, therefore, we compared the prognostic power of NT-proBNP, cTnT, and hsCRP for CV and all-cause mortality in incident Korean hemodialysis (HD) patients from the Clinical Research Center for ESRD (CRC for ESRD) cohort. Moreover, the relationship between these biomarkers and echocardiographic parameters were elucidated.

## SUBJECTS AND METHODS

### Patients

All ESRD patients who started HD between August 1, 2009 and February 29, 2012 at 36 centers of the CRC for ESRD in Korea were initially recruited for this prospective observational multicenter study. We excluded patients who were younger than 18 years old, had histories of peritoneal dialysis or kidney transplantation before HD, had underlying active malignancy, or were expected to survive less than 3 months. Patients who died within 3 months after the commencement of HD were also excluded from the study. Finally, a total of 864 incident HD patients were included in the final analysis.

The study protocol was approved by the Institutional Review Boards of each participating center and all patients provided their written informed consent to participate in the study.

### Data Collection

Demographic and clinical data at the time of study entry, including age, gender, body mass index (BMI) calculated as weight/height,^2^ primary renal disease, comorbidities, and medications, were recorded. Coronary arterial disease (CAD) was defined as a history of angioplasty, coronary artery bypass grafts, myocardial infarction, or angina, while peripheral arterial disease (PAD) was defined as a history of claudication, ischemic limb loss, and/or ulceration, or peripheral revascularization. The following laboratory data were measured from predialysis fasting blood samples taken on the day of the midweek dialysis session close to the time of discharge, when the patients were considered to be clinically stable and in a euvolemic state: hemoglobin (Hb), white blood cell (WBC) count, blood urea nitrogen, creatinine, calcium, phosphorus, intact parathyroid hormone (iPTH), albumin, total cholesterol, triglycerides, sodium, potassium, bicarbonate, serum iron, ferritin, NT-proBNP, cTnT, and hsCRP. Since measurement of cTnI was not standardized or available in some centers of the CRC for ESRD, cTnT was used as a marker for cardiac troponin.

NT-proBNP and cTnT concentrations were determined using the Elecsys proBNP electrochemiluminescence immunoassay (Roche Diagnostics, Indianapolis, IN) and a third-generation electrochemiluminescence immunoassay (Elecsys Troponin T STAT Immunoassay, Roche Diagnostics), respectively, while hsCRP levels were measured by a latex-enhanced immunonephelometric method using a BNII analyzer (Dade Behring, Newark, DE).

### Echocardiography and Electrocardiogram

Echocardiography was performed on a non-dialysis day close to the time of discharge based on the imaging protocol recommended by the American Society of Echocardiography,^29^ to assess the volume status and/or cardiac function of the patients. Even though the timing for echocardiography was not standardized, it was mainly performed on the day after the last or 2nd last HD performed during admission. Left atrial dimension (LAD) was assessed at end-ventricular systole at the level of aortic valve according to the leading-edge-to-leading-edge convention. Left ventricular mass (LVM) was determined using the method described by Devereux et al^30^ and the LV mass index (LVMI) was calculated by dividing LVM by body surface area. LV systolic function was defined by LV ejection fraction (LVEF) using a modified biplane Simpson's method from the apical 2-chamber and 4-chamber views. We chose and measured right ventricular systolic pressure (RVSP) to evaluate the volume status of our patients. RVSP was measured by continuous wave Doppler echocardiography using the modified Bernoulli equation (*p* = 4 × *v*^2^ + right atrial pressure, where *v* = the peak tricuspid regurgitant velocity and right atrial pressure was assumed to be 10 mm Hg). Multiple reproducibility, inter-reader reliability, intrareader reliability, and reader drift analyses were performed at a core echocardiography laboratory (Kyungpook National University) on a 3% random sample of the entire cohort each year. The intraclass correlation coefficients for the echocardiographic measures were 0.773 for LAD, 0.745 for LVMI, 0.842 for LVEF, and 0.787 for RVSP. Furthermore, products of QRS duration multiplied by the Cornell voltage combination (with 6 mm added in women) ≥2440 mm ms were used to determine LVH on electrocardiogram (ECG).^31^

### Outcome Measures

For the current study, all mortality events were retrieved from the database and carefully reviewed. The primary and secondary endpoints were CV and all-cause mortality, respectively. CV mortality was considered death from myocardial infarction or ischemia, congestive heart failure, pulmonary edema, and cerebral hemorrhage or vascular disorder.

### Statistical Analysis

Statistical analyses were performed using SPSS for Windows, version 18.0 (SPSS Inc., Chicago, IL). Continuous variables were expressed as mean ± standard deviation and categorical variables as a number (percentage). Patients were dichotomized into “high” and “low” groups based on the median values of NT-proBNP, cTnT, and hsCRP, and the baseline characteristics were compared between the 2 groups using Student's *t*-test for continuous variables and the chi-square test for categorical variables. Because the distributions of NT-proBNP, cTnT, and hsCRP concentrations were log-normal, natural log values (Ln) were used in the analysis. Cumulative survival curves were generated by the Kaplan–Meier method, and between-group survival was compared by a log-rank test. In addition, multivariate regression analyses with traditional risk factors and each cardiac biomarker were conducted, and the discrimination power of each multivariate model was compared to assess the additional impact of each biomarker to traditional risk factors. For a null model, we applied backward method (specifies the significance level for entering effects = 0.05 and removing effects = 0.05) on a candidate list of traditional risk factors, including age, gender, hypertension, diabetes mellitus (DM), Charlson comorbidity index (CCI), mean arterial pressure (MAP), 24-hour urine output, hemoglobin (Hb), and serum albumin and total cholesterol, and found that only age was a significant variable for CV and/or all-cause mortality. Therefore, it was inevitable to choose age, gender, hypertension, and DM, well-known traditional risk factors of CVD, as variables of a null model. Meanwhile, since CCI had a strong association with age (*γ* = 0.614, *P* < 0.001), only age was entered into the model to avoid multicollinearity. Moreover, time-dependent receiver operating characteristic (ROC) curve was constructed to assess which cardiac biomarkers added the higher prognostic value.^32,33^ We especially compared the global concordance probability (integrated area under the curve, iAUC) between traditional risk factors and each cardiac biomarker by using The R Statistical package ver. 3.0.1 (www.R-project.org). Furthermore, we calculated the net reclassification index (NRI) and the integrated discrimination improvement (IDI) to assess the ability of the models with biomarkers to correctly reclassify patients compared to the model without biomarkers (model including traditional risk factors and each cardiac biomarker). The NRI required the definition of risk strata. We defined 3 risk strata for CV and all-cause mortality based on 3 points (<33.3%, 33.3–66.6%, and >66.6%). In the NRI, only the changes in predicted probabilities that imply a change from 1 category to another were considered. Therefore, the NRI expressed the global net improvement in reclassification with the new model. By contrast, the IDI did not require a prior definition of risk strata, thus considering the change in the predicted probabilities as a continuous variable.^34^*P*-values less than 0.05 were considered statistically significant.

## RESULTS

### Patient Characteristics

The baseline patient characteristics are shown in Table 1 . The mean age was 59.7 ± 14.4 years old, and 513 patients (59.4%) were male. The most common comorbid disease was DM (56.3%), followed by hypertension (48.0%). The median value of NT-proBNP, cTnT, and hsCRP were 6019.5 pg/mL, 0.05 ng/mL, and 0.34 mg/dL, respectively. The mean values of LAD, LVMI, LVEF, and RVSP were 4.2 cm, 185.4 g/m^2^, 58.5%, and 33.8 mm Hg, respectively. In addition, LVH on ECG was present in 264 patients (30.6%). The mean duration of follow-up by a nephrologist before commencing dialysis was 10.2 months. Most patients were prescribed with low flux at the time of HD initiation. However, 2.5% and 2.0% of this study subjects used high flux and hemodiafiltration at the start time of HD. Dialysis mode was changed in 152 patients (17.6%) during study period, and most of the change was from low flux to high flux (131/152 [86.2%] episodes). Six hundred and thirteen patients (71.0%) used temporary catheter, and native vascular access was available in only 206 patients (23.8%). Acute HD was required in 485 patients (56.1%).

**TABLE 1:**
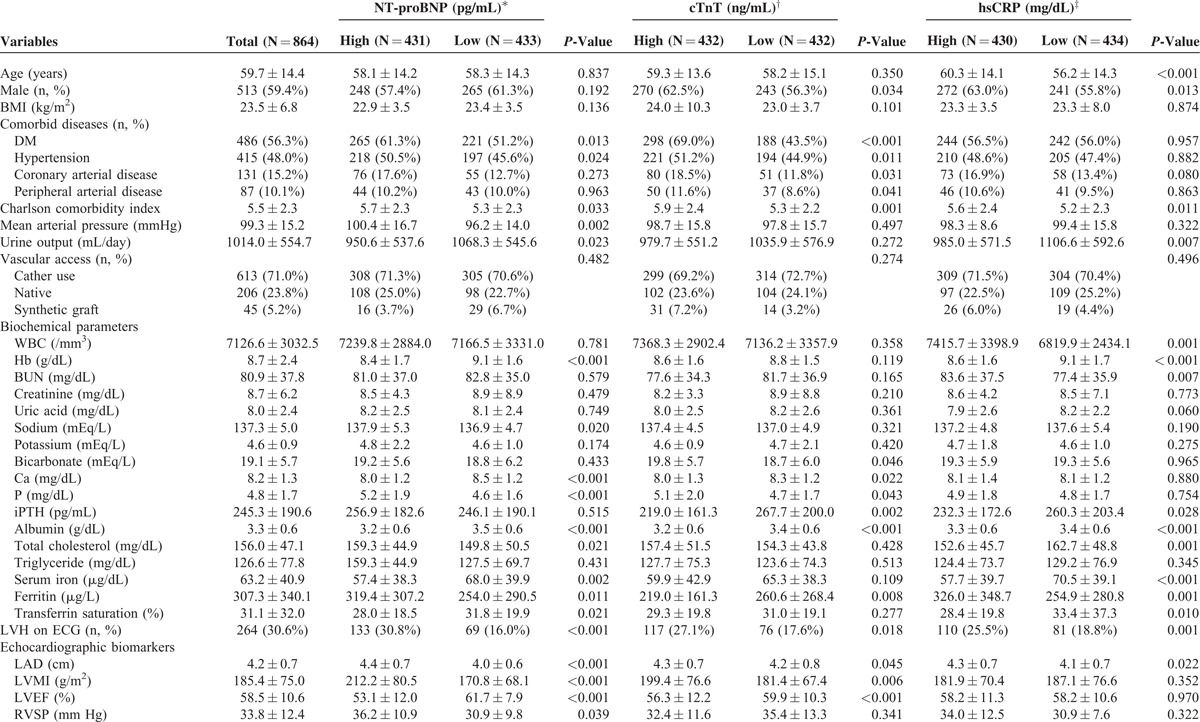
Baseline Clinical Characteristics and Biomarkers of the Study Population

**TABLE 1 (Continued):**
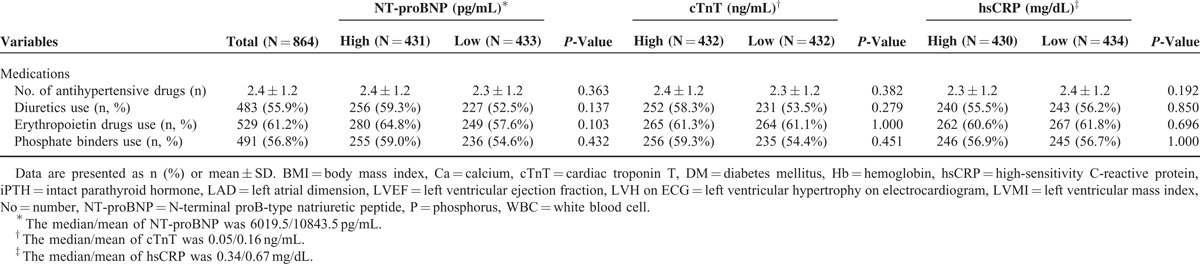
Baseline Clinical Characteristics and Biomarkers of the Study Population

First, the baseline patient characteristics were compared between the 2 dichotomized groups based on the median concentrations of NT-proBNP, cTnT, and hsCRP. DM and hypertension were significantly more prevalent in the “high” NT-proBNP and cTnT groups compared to the “low” NT-proBNP and cTnT groups, respectively, but there was no significant difference in the proportion of patients with DM or hypertension between the “high” and “low” hsCRP groups. Moreover, the proportions of patients with CAD or PAD were significantly higher in the “high” cTnT group. The CCI was significantly higher and serum albumin levels were significantly lower in the “high” NT-proBNP, cTnT, and hsCRP groups compared to their corresponding “low” groups. LVH on ECG was also significantly more prevalent in all 3 “high” groups. Whereas all “high” biomarker groups had significantly higher mean values of LAD, LVMI was significantly higher and LVEF was significantly lower only in the “high” NT-proBNP and cTnT groups, but not in the “high” hsCRP group. Moreover, RVSP was significantly higher in patients with “high” NT-proBNP compared to the “low” NT-proBNP (36.2 vs 30.9 mm Hg, *P* = 0.039), while there were no significant differences in RVSP between the “high” and “low” cTnT and hsCRP groups. In contrast, WBC counts were significantly higher in the “high” hsCRP group compared to the “low” hsCRP group (Table 1 ).

Next, we compared CV and all-cause mortality between the “high” and “low” biomarker groups. Even though there were significant differences in all-cause mortality between all 3 “high” and “low” biomarker groups (NT-proBNP, 11.1% vs 3.7%, *P* = 0.007; cTnT, 10.0% vs 4.9%, *P* = 0.045; and hsCRP, 9.7% vs 5.1%, *P* = 0.019), CV mortality was significantly higher only in the “high” NT-proBNP (5.8% vs 0.7%, *P* = 0.003) and cTnT groups (4.9% vs 1.6%, *P* = 0.027), but not in the “high” hsCRP group (4.4% vs 2.1%, *P* = 0.198) (Table 2). In additional analysis, we determined the cut-off point for cTnT value based on the ROC curve, and it was revealed to be 0.045 ng/mL, which is similar to the median level of 0.05 ng/mL. When we divided the patients into 2 groups (high vs low group) according to the cut-off value for cTnT and compared all-cause and CV mortality rates, both of them were significantly higher in the “high” cTnT group compared with the “low” cTnT group (all-cause mortality, 12.0 vs 1.8%, *P* = 0.001; CV mortality; 5.7 vs 0.2%, *P* = 0.004) (Supplementary Table 1, http://links.lww.com/MD/A92).

**TABLE 2:**
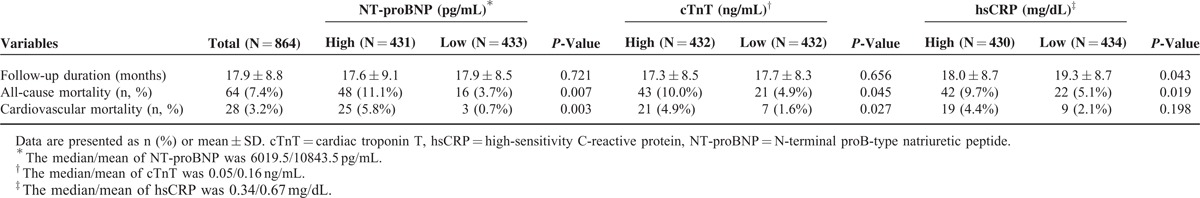
Comparisons of Clinical Outcomes Between Each Group Stratified Based on the Median Value of Cardiac Biomarkers

### Clinical Outcomes Based on Biomarker Levels

**FIGURE 1:**
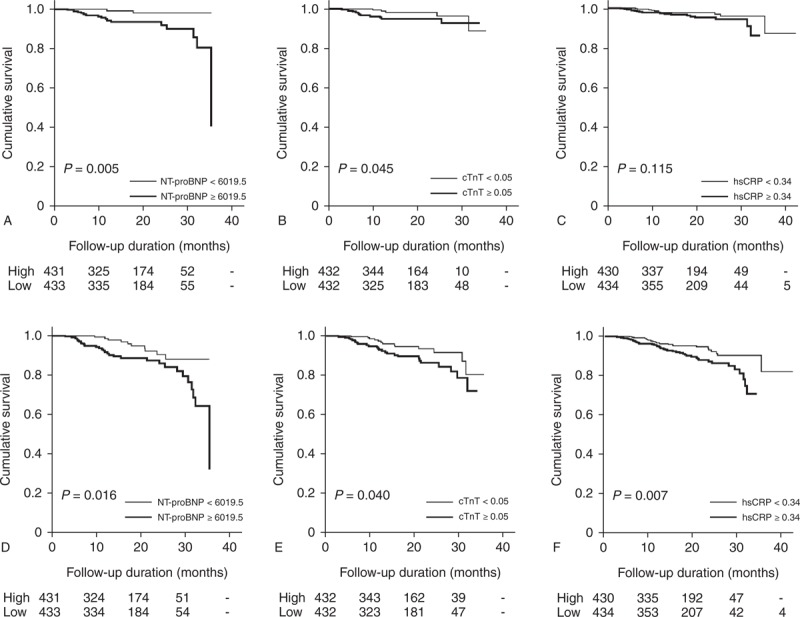
Kaplan–Meier survival curves for cardiovascular and all-cause mortality based on the median baseline values of NT-proBNP (A/D), cTnT (B/E), and hsCRP (C/F). The CV survival rates were significantly lower in the “high” NT-proBNP and cTnT groups compared to the corresponding “low” groups, while there was no significant difference in CV survival rates between the “high” and “low” hsCRP groups (A, B, and C). However, the all-cause mortality rates were significantly higher in all 3 “high” groups (D, E, and F). cTnT = cardiac troponin T, CV = cardiovascular, hsCRP = high-sensitivity C-reactive protein, NT-proBNP = N-terminal proB-type natriuretic peptide.

During a mean follow-up duration of 17.9 ± 8.8 months, 64 patients (7.4%) died. Among them, 28 patients (48.3%) died from CV causes. As shown in Figure 1, the CV survival rates were significantly lower in the “high” NT-proBNP (*P* = 0.005) and cTnT groups (*P* = 0.045) compared to the corresponding “low” groups, while there was no significant difference in CV survival rates between the “high” and “low” hsCRP groups (*P* = 0.115). However, the all-cause mortality rates were significantly higher in all 3 “high” groups (NT-proBNP, *P* = 0.016; cTnT, *P* = 0.040; and hsCRP, *P* = 0.007).

**FIGURE 2:**
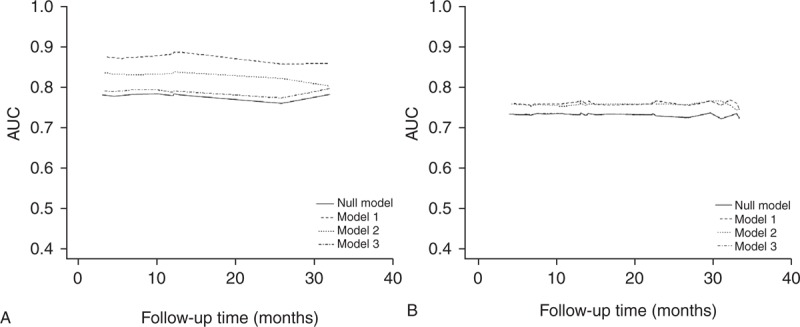
Time-dependent ROC curve analyses for cardiovascular (A) and all-cause mortality (B). iAUC values for CV mortality were 0.815 (95% CI, 0.701–0.937) for traditional risk factors, and 0.897 (95% CI, 0.794–0.984) for traditional risk factors with Ln NT-proBNP. The ESD in iAUC was 0.083 (95% CI, 0.015–0.171). In addition, iAUC values for all-cause mortality were 0.748 (95% CI, 0.655–0.828) for traditional risk factors, and 0.778 (95% CI, 0.684–0.862) for traditional risk factors with Ln NT-proBNP, and the ESD in iAUC value for all-cause mortality was 0.031 (95% CI, 0.001–0.088). Regarding cTnT, the ESDs in iAUC for CV and all-cause mortality were 0.054 (95% CI, 0.008–0.113) and 0.026 (95% CI, 0.001–0.079), respectively. However, the ESDs of hsCRP for CV and all-cause mortality were 0.017 (95% CI, −0.003 to 0.077) and 0.006 (95% CI, −0.003 to 0.034), respectively. ^∗^Null model; including traditional risk factors, such as age, gender, hypertension, and DM. ^∗∗^Model 1; Null model plus Ln NT-proBNP. ^∗∗∗^Model 2; Null model plus Ln cTnT. ^∗∗∗^Model 3; Null model plus Ln hsCRP. CI = confidence interval, cTnT = cardiac troponin T, CV = cardiovascular, hsCRP = high-sensitivity C-reactive protein, iAUC = integrated area under curve, NT-proBNP = N-terminal proB-type natriuretic peptide, ROC = receiver operating curve.

Time-dependent ROC curves over the entire follow-up period are presented in Figure 2. iAUC values for CV mortality were 0.815 (95% CI, 0.701–0.937) for traditional risk factors (including age, gender, hypertension, and DM), and 0.897 (95% CI, 0.794–0.984) for traditional risk factors with Ln NT-proBNP. The estimated difference (ESD) in iAUC was 0.083 (95% CI, 0.015–0.171), indicating that NT-proBNP was an additional significant prognostic factor for CV mortality. In addition, iAUC values for all-cause mortality were 0.748 (95% CI, 0.655–0.828) for traditional risk factors, and 0.778 (95% CI, 0.684–0.862) for traditional risk factors with Ln NT-proBNP, and the ESD in iAUC value for all-cause mortality was 0.031 (95% CI, 0.001–0.088), suggesting that NT-proBNP was also an additional significant prognostic factor for all-cause mortality. Regarding cTnT, the ESDs in iAUC for CV and all-cause mortality were 0.054 (95% CI, 0.008–0.113) and 0.026 (95% CI, 0.001–0.079), respectively, which represented that cTnT was also an additional significant useful prognostic factor for CV and all-cause mortality. However, the ESDs of hsCRP for CV and all-cause mortality were 0.017 (95% CI, −0.003 to 0.077) and 0.006 (95% CI, −0.003 to 0.034), respectively.

### Biomarkers as Predictors of Mortality

Univariate Cox proportional hazard regression analysis revealed that Ln NT-proBNP and Ln cTnT but not Ln hsCRP were associated with a higher risk of CV (Ln NT-proBNP, HR = 2.228, *P* = 0.002; Ln cTnT, HR = 1.274, *P* = 0.015) and all-cause mortality (Ln NT-proBNP, HR = 1.392, *P* = 0.012; Ln cTnT, HR = 1.182, *P* = 0.034). Moreover, age and LVMI were found to be significant predictors of CV and all-cause mortality. However, hypertension was demonstrated to be associated with all-cause mortality but not with CV mortality, while DM and LVEF were revealed as significant risk factors only for CV mortality (Table 3). In multivariate regression analyses, NT-proBNP (CV, HR = 2.236 [1.304–3.831], *P* = 0.003; and all-cause, HR = 1.361 [1.034–1.793], *P* = 0.028) was still found as a significant independent risk factor for CV and all-cause mortality even after adjustment for traditional risk factors, whereas cTnT and hsCRP were not significant prognostic factors (Tables 4 and Tables 5). Furthermore, we calculated the NRI and the IDI to assess the ability of the models with biomarkers to correctly reclassify patients compared to the model without biomarkers (model including traditional risk factors and each cardiac biomarker). The prognostic powers for null and each cardiac biomarker model are shown in Table 6. There were significant differences between null model (including traditional risk factors) and null model plus NT-proBNP or cTnT, but not hsCRP. However, NT-proBNP was a more prognostic marker for CV and all-cause mortality compared to cTnT.

**TABLE 3:**
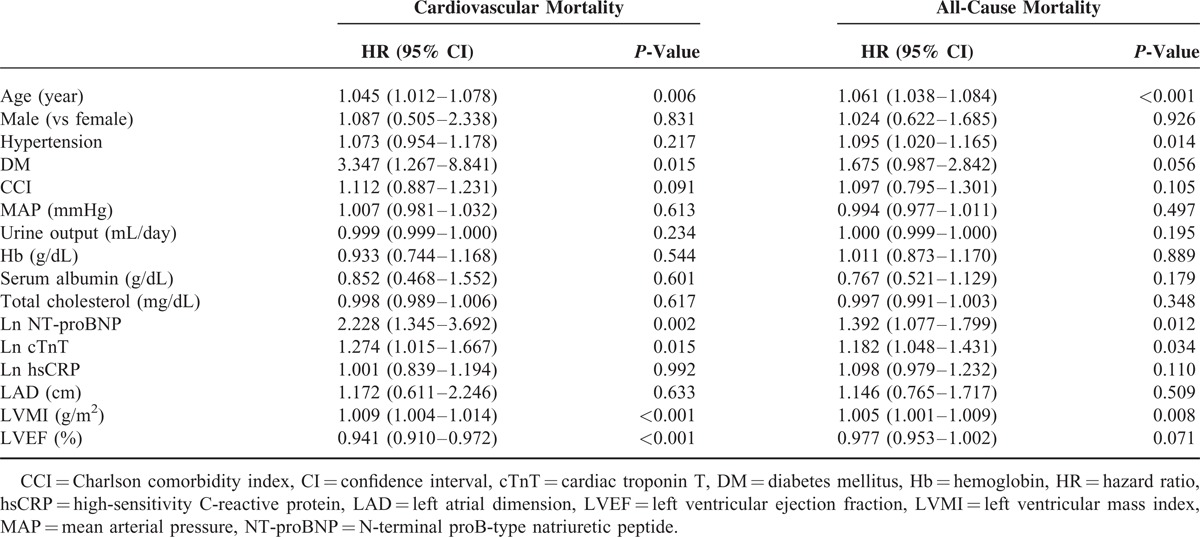
Univariate Cox Proportional Regression Analysis for Cardiovascular and All-Cause Mortality

**TABLE 4:**
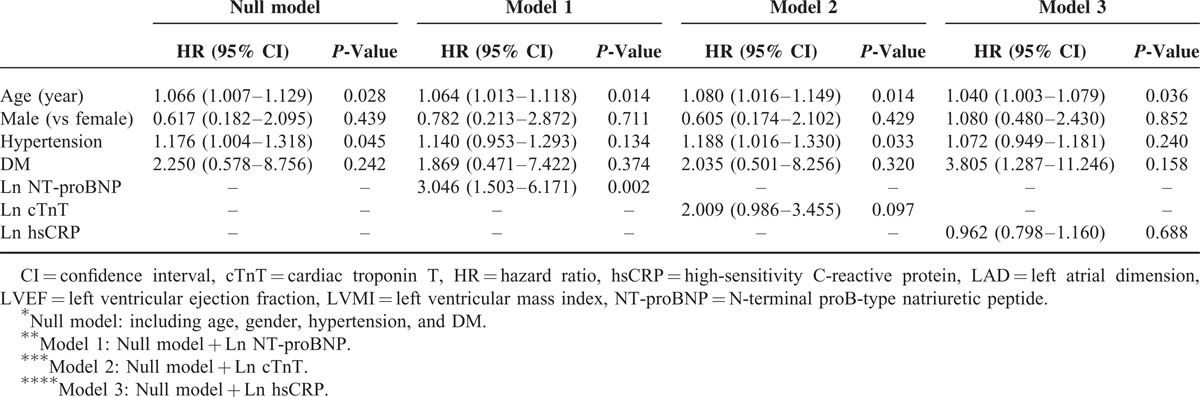
Multivariate Cox Proportional Regression Analysis for Cardiovascular Mortality

**TABLE 5:**
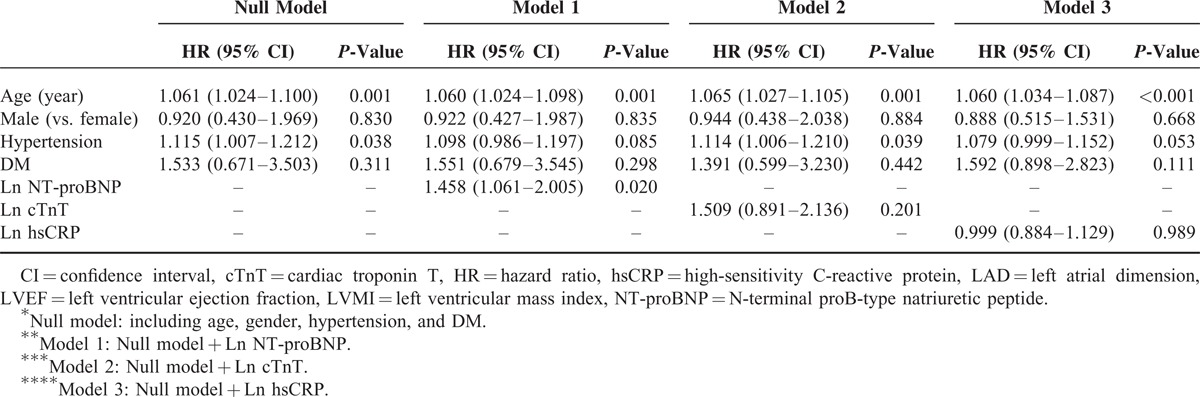
Multivariate Cox Proportional Regression Analysis for All-Cause Mortality

**TABLE 6:**
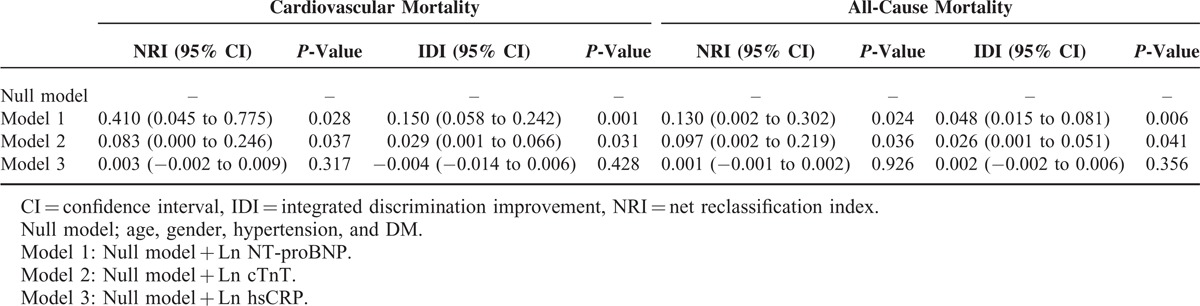
Prognostic Power for Cardiovascular and All-Cause Mortality for Null and Each Cardiac Biomarker Models Using NRI and IDI

## DISCUSSION

Even though a number of previous studies have found that some biochemical markers, such as NT-proBNP, cTnT, and hsCRP, predict CV mortality in ESRD patients, most of the patients included in these studies were prevalent dialysis patients.^10,11,23^ The results of the present study demonstrate that NT-proBNP concentration, but neither cTnT nor hsCRP, is independently associated with CV and all-cause mortality and is a more prognostic marker for CV and all-cause mortality compared to cTnT. To our knowledge, the present study is the first study to investigate and to compare the impact of various biomarkers at the time of dialysis initiation on CV and all-cause mortality in a large, ethnically homogeneous, incident HD patient cohort.

Cardiac biomarkers, including BNP, NT-proBNP, cTnT, and cTnI, have been considered to represent the current status and function of the heart.^15–21^ In addition, a number of previous studies have shown that these cardiac biomarkers are useful in defining CV risk stratification in the general population and in patients with specific diseases.^8,10,11,22,23^ Moreover, the biology, pathophysiology, and the prognostic value of these biomarkers in ESRD patients have already been extensively reviewed by Angela and Lai.^8^ In accordance with most previous studies on prevalent ESRD patients, we also found that NT-proBNP was an independent risk factor for CV and all-cause mortality in incident HD patients. Moreover, the prognostic value of NT-proBNP for CV and all-cause mortality was somewhat higher than that of cTnT, which was in concordance with the study by Satyan et al.^10^ The reason for this better predictive power of NT-proBNP has not been fully explained, but one possibility is that NT-proBNP is more closely associated with LVH, which is known to have a great impact on CV and all-cause mortality in ESRD patients.^10,18,19,23^

LVH is a well-known powerful independent predictor of CV mortality in patients with ESRD.^35–38^ Moreover, the change in LVH has been demonstrated as a strong prognostic factor in these patients.^36,37^ A previous prospective study on prevalent HD patients revealed that the rates of LVMI increase were significantly higher in patients with incident CV events than in those without such events and that cardiovascular event-free survival in patients with changes in LVMI below the 25th percentile was significantly higher than in those with changes above the 75th percentile.^36,39^ Similarly, in a cohort study of 153 incident ESRD patients receiving HD, a 10% reduction in LVM during a mean follow-up duration of 54 months resulted in a 28% decrease in CV mortality and a 22% decrease in all-cause mortality.^37,39^ In that study, LVM regression was also independently associated with improved patient survival even after adjustment for age, gender, diabetes, history of CVD, and all nonspecific CV risk factors.^37^ While these 2 studies used echocardiography to assess LVMI or LVM as an indicator of LVH, similar results were observed in hypertensive patients with LVH on ECG.^40,41^ In the current study, the differences in the proportion of patients with LVH on ECG and LVMI values were most prominent between “high” and “low” NT-proBNP groups. Furthermore, NT-proBNP but neither cTnT nor hsCRP significantly correlated with LVMI. In addition, most previous studies used LVMI, assessed by echocardiography, as an indicator of LVH. Meanwhile, since NT-proBNP synthesis and secretion are mainly related to increased LV wall stress, circulating NT-proBNP levels are considered to reflect the degree of LV overload. Taken together, we surmised that the best prognostic value of NT-proBNP was attributed to its strong association with LVH.

In this study, the median levels of cTnT (0.05 ng/mL) were only one-half of the reference cTnT concentrations (0.1 ng/mL) used in most previous studies,^12,42,43^ while the median NT-proBNP levels were comparable to other studies.^10,11,23^ In addition, even though the mean NT-proBNP concentrations were significantly lower in the “low” cTnT group compared to the “high” cTnT group (9524 vs 20,927 pg/mL, *P* < 0.001), the mean NT-proBNP levels in the “low” cTnT group were regarded as significant concentrations in previous studies.^10,11,23^ These findings may in part contribute to the lower predictive power of cTnT for mortality than NT-proBNP.

Moreover, the proportion of patients with preexisting CAD and PAD was significantly higher in the “high” cTnT group compared to the “low” cTnT group. In the future, clarification is needed to determine whether a weaker association between cTnT and mortality can be attributed to more meticulous care and more intensive treatment received by these patients.

hsCRP, an acute phase reactant, has been considered a marker of inflammation.^24–26^ Since accumulating evidence indicates that inflammation is an integral part of the development and progression of atherosclerosis, it has been proposed that hsCRP levels are closely linked with the presence of CVD.^24–26^ Furthermore, numerous studies have found that the serum concentration of hsCRP is predictive of CV mortality as well as future CV events in the general population.^26^ However, the results of previous studies on the association between hsCRP levels and CVD or CV mortality in ESRD patients are not consistent.^11,23^ These conflicting results may be due to prevalent chronic low-grade inflammation in ESRD patients.^44^ Especially in HD patients, extracorporeal circulation of blood, bioincompatible dialyzer, and non-sterile dialysate and back leak of dialysate may lead to a state of chronic inflammation.^45–47^ In addition, hsCRP levels can be elevated by diabetes, insulin resistance, and dyslipidemia, all of which are frequently observed in ESRD patients on HD.^14,18^ Moreover, hsCRP concentrations are reported to vary widely, both intra- and interindividually.^48,49^ Therefore, the prognostic power of hsCRP for CV mortality could be lessened in ESRD patients. The results of the present study, demonstrating the lack of an association of hsCRP with CV mortality can be interpreted in this point of view. Meanwhile, we showed that the all-cause mortality was significantly more prevalent in the “high” hsCRP group compared to the “low” hsCRP group. The Kaplan–Meier plot also revealed that all-cause mortality rates were significantly higher in the “high” hsCRP group. Since WBC counts and serum ferritin levels were significantly higher in the “high” hsCRP group compared to the “low” hsCRP group, we inferred that infection-related death may account for this higher all-cause mortality in the “high” hsCRP group. Due to a relatively small number of deaths from infection, however, it was difficult to analyze the association between hsCRP and infection-related mortality. By the same token, the independent predictability of hsCRP for all-cause mortality might not be significant.

There are several limitations of this study. First, since the study subjects were all Korean incident HD patients, the associations between various biomarkers and mortality may not be generalizable to other populations. Second, all biomarker measurements and echocardiography were performed only once during the admission for HD commencement; therefore, it was difficult to clarify why some but not all biomarkers had associations with mortality and to demonstrate the impact of the changes in these biomarkers on patients’ clinical outcomes. Future studies will be necessary to find out whether the changes in biomarkers over time have an association with the clinical outcomes. Third, CV and all-cause mortality rates in the current study were lower compared to those in previous studies on Western ESRD patients.^10,11,23^ We hypothesize that the difference was mainly attributed to disparate ethnicities, because the mortality rates of our patients were comparable to those of Japanese patients on HD.^50^ Fourth, we arbitrarily stratified the patients based on the median values of cardiac and inflammatory biomarkers. Previous studies, which investigated the impact of biomarkers on CV outcomes in ESRD patients, also used very diverse cut-off values for these biomarkers.^10,11,22,23^ Therefore, it is necessary to define the best cut-off concentrations of each biomarker in both HD and peritoneal dialysis patients. In additional analyses, the clinical outcomes were compared after stratifying these patients into tertiles (lower, middle, and upper groups) according to the baseline NT-proBNP, cTnT, and hsCRP concentrations. During the follow-up period, patients in the upper tertile of NT-proBNP and cTnT exhibited significantly higher CV and all-cause mortality rates compared to those in other tertiles (*P* = 0.011 and *P* = 0.005 for NT-proBNP, and *P* = 0.046 and *P* = 0.041 for cTnT). On the contrary, only all-cause mortality rates in the upper tertile of hsCRP were significantly higher than those in other tertiles (*P* = 0.017), while there was just a trend for an increase in CV mortality in patients in the upper tertile of hsCRP (*P* = 0.292). Meanwhile, Shafi et al^10^ also suggested that a stratification approach based on cTnI and NT-proBNP levels could be useful to control blood pressure properly in hemodialysis patients. Taken together, not only cTnT but also cTnI may be associated with worse clinical outcomes in incident HD patients. Fifth, the follow-up duration was short in this study. Even though the follow-up duration seems to be relatively short, these patients have continuously been followed up and thus a better long-term study will be carried out in a near future. Moreover, there were not a few follow-up losses, which could lessen the statistical power, but it was not easy to find out the exact reasons for them. Therefore, the results of this study should be interpreted with caution. We also regarded this situation as one of the limitations of the present study. Sixth, during the follow-up duration, all-cause mortality occurred in 64 patients, whereas only 28 patients died of CV events. Even though the number of variables in a null model might be suitable for analyzing all-cause mortality, we surmised that the risk of overfitting could be run when all the variables in a null model was applied for investigating CV mortality. Therefore, a further study with a long-term follow-up duration is needed to verify our results. Seventh, the measurement of biomarkers was not made with immediate post-dialysis blood samples when clinical euvolemia was reached. Considering the results of some previous studies showing that post-dialysis hormone levels may vary significantly,^51^ there is a possibility that the levels of biomarkers can be influenced by patients’ hydration status. Therefore, if more than one measurement in separate dialysis session was performed, the results might be even more accurate. Furthermore, the current study did not perform an objective fluid balance monitoring, such as inferior vena cava diameter, bioimpedance, and continuous blood volume measurements. In this cohort study, however, routine chest X-rays and physical examination were performed to evaluate the volume status of these patients, and these cardiac biomarkers were determined close to time of discharge, when the physicians considered their patients to be clinically euvolemic. Since all laboratory data including NT-proBNP were compared to each other in this study, we presumed that measurements of these laboratory parameters on different days might be regarded rather as a more serious issue. In addition, target dry weights were established for each patient totally based on their physicians’ judgment. Therefore, we could not completely discriminate among hypovolemic, euvolemic, and hypervolemic patients after dialysis. However, since the laboratory measurements and echocardiography were performed close to the time of discharge, we inferred that a majority of patients were euvolemic at postdialysis. Despite these limitations, to our knowledge, the present study is the first study to investigate and to compare the association of NT-proBNP, cTnT, and hsCRP levels at the time of dialysis initiation with CV and all-cause mortality in a large, ethnically homogeneous, incident HD patient cohort. Further studies are needed to clarify whether the concentrations of these biomarkers can provide a guideline for treating ESRD patients and whether serial monitoring rather than a single measurement of biomarkers is helpful in identifying ESRD patients at a high risk of CV mortality.

In conclusion, NT-proBNP is the biomarker that results in the most added prognostic value on top of traditional risk factors for CV and all-cause mortality in incident HD patients.
